# Supplementary material for: Identification of photoperiod-induced specific miRNAs in the adrenal glands of Sunite sheep (Ovis aries)
Source: Front Vet Sci. 2022 Jul 22;9:888207. doi: 10.3389/fvets.2022.888207 (PMC9354845; doi:10.3389/fvets.2022.888207)
Supplement: Supplementary file 1 [file Data_Sheet_1.ZIP › Supplementary file/miRDeep2 file.zip!/miRDeep2 file/Novel_Structure/SPLP42A3/Novel_14.pdf]

Provisional ID	:	NC_019459.2_4430
Score total	:	20.4
Score for star read(s)	:	3.9
Score for read counts	:	12.9
Score for mfe	:	2.1
Score for randfold	:	1.6
Score for cons. seed	:	
Total read count	:	37
Mature read count	:	33
Loop read count	:	0
Star read count	:	4

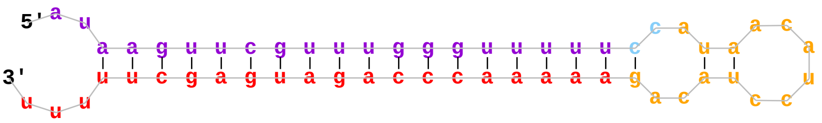

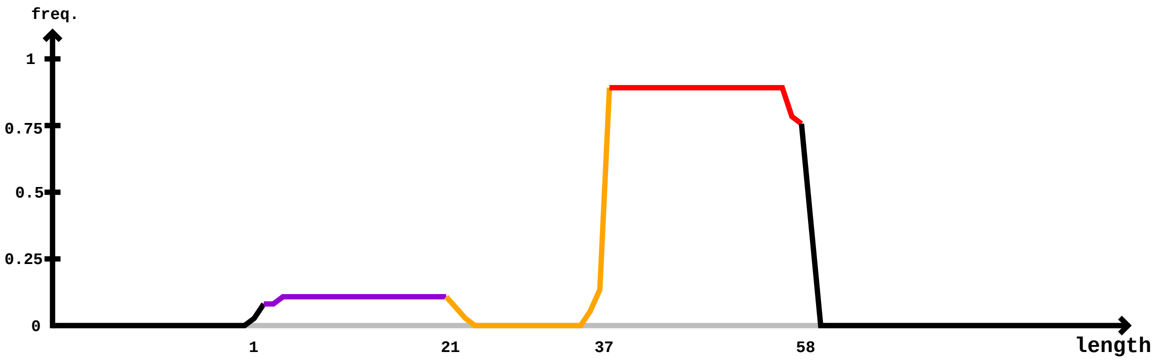

Star **Mature**

```
5'- aauacauguuggaguggccaauaaguucguuuggguuuuuccauaacauccuacagaaaaacccagaugagcuuuuuggccaacccaauacuuuauuuccuuuucggguc   -3'    obs
    aauacauguuggaguggccaauaaguucguuuggguuuuuccauaacauccuacagaaaaacccagaugagcuuuuuggccaacccaauacuuuauuuccuuuucggguc          exp
    ......(((((..((((((((.((((((((((((((((((..((......))..))))))))))))))))))).))))))))..)))))........(((....)))..   reads     mm      sample
    ...................aauaaguucguuuggguuuuu......................................................................   1         0       seq
    ....................auaaguucguuuggguuuuu......................................................................   2         0       seq
    ......................aaguucAuuuggguuuuucc....................................................................   1         1       seq
    ......................................................agaaaaacccagaugaAcuuu...................................   2         1       seq
    .......................................................gaaaaacccagaugaAcuuu...................................   2         1       seq
    .......................................................gaaaaacccagaugaAcuuuuu.................................   1         1       seq
    ........................................................aaaaacccagaugaAcuuuu..................................   1         1       seq
    ........................................................aaaaacccagaugaAcuuuuu.................................   27        1       seq
```
